# Microscale heat transfer and thermal extinction of a wire-grid polarizer

**DOI:** 10.1038/s41598-018-33347-5

**Published:** 2018-10-08

**Authors:** Seongmin Im, Eunji Sim, Donghyun Kim

**Affiliations:** 1 School of Electrical and Electronic Engineering Yonsei University, Seoul, 03722 Korea; 2 Department of Chemistry, Yonsei University, Seoul, 03722 Korea

## Abstract

We explore heat transfer and thermal characteristics of a wire-grid polarizer (WGP) on a microscale by investigating the effect of various geometrical parameters such as wire-grid period, height, and a fill factor. The thermal properties arise from heat transfer by light absorption and conduction in wire-grids. Fill factor was found to be the most dominant geometrical parameter. For TM polarized light, a higher fill factor with thicker wire-grids increased the temperature. The local temperature was found to rise up to T_max_ = 354.5 K. TE polarization tended to produce lower temperature. Thermal extinction due to polarimetric extinction by a WGP was also evaluated and highest extinction was observed to be 4.78 dB, which represents a temperature difference ΔT = 54.3 °C. We expect the results to be useful for WGPs in polarization-sensitive thermal switching applications.

## Introduction

A wire-grid polarizer (WGP) is an optical element consisting of wire-grids: TE polarized light with an electric field aligned in an orientation parallel to the wire-grids drives a conduction current and produces Joule heating in the wire (absorption) or re-radiation in the backward direction (reflection)^1^. On the other hand, TM polarized light can propagate through the grids, thereby transmitted or reflected light becomes strongly polarized. A WGP has drawn significant interests, initially as an infrared polarizing element^2^, because of the excellent polarization performance combined with planar structure that allows an array-type polarization filter and easy integration to other optical components. For example, a WGP has been integrated to a photodiode^3^, liquid crystal^4–7^, fiber-optics^8^, optical isolators^9,10^, semiconductor laser^11^, light-emitting diodes^12^, contact lens^13^, photoelectrochemical solar cells^14^, and a CMOS and CCD imaging sensor^15–19^. A WGP has found applications in imaging^20^ and spectro-polarimetry^21,22^ and also in projection displays^23^. Polarimetry using a biomolecular WGP consisting of hybridizing DNA-nanoparticle composites was reported^24^. In addition, effects of surface roughness were analyzed on WGP performance^25^. A WGP was explored on a rotating platform^26^ and flexible surface^27–30^, while a plasmonic WGP was investigated based on blazed grating structure^31^. Significant part of WGP research has largely been limited to fabrication issues, such as fabrication of WGPs by way of nanoimprinting^32–37^. Simplified fabrication due to longer wire-grid period requirement allows applications in microwave and terahertz wavebands^30,38,39^.

In all these studies, thermal characteristics produced by the energy distribution of light fields have been hardly of interest, as the most attention has been paid to evaluating and optimizing polarimetric performance in the far-field, which is measured by

1 $$T{r}^{TE}=\frac{\langle {E}_{t}^{TE}\times {H}_{t}^{TE}\rangle }{\langle {E}_{i}^{TE}\times {H}_{i}^{TE}\rangle }$$

2 $$T{r}^{TM}=\frac{\langle {E}_{t}^{TM}\times {H}_{t}^{TM}\rangle }{\langle {E}_{i}^{TM}\times {H}_{i}^{TM}\rangle }$$

3 $$ER=|\frac{\langle {E}_{t}^{TM}\times {H}_{t}^{TM}\rangle -\langle {E}_{t}^{TE}\times {H}_{t}^{TE}\rangle }{\langle {E}_{i}^{TM}\times {H}_{i}^{TM}\rangle +\langle {E}_{i}^{TE}\times {H}_{i}^{TE}\rangle }|.$$

Here, *Tr* represents transmittance of TE and TM polarization. *ER* is an extinction ratio of TM over TE polarization. *ER* ranges from 0 (no polarization) to 1 (perfect polarization) and is often defined otherwise as *ER* = *Tr*^*TM*^/*Tr*^*TE*^ with 1 for no polarization and ∞ for perfect polarization. *E*_*i*_ and *E*_*t*_ (*H*_*i*_ and *H*_*t*_) denote electric (magnetic) field amplitude of incident and transmitted light. 〈 〉 refers to a time and space average of light fields in the far-field. Inside the bracket is, in fact, the Poynting vector of a light field. As WGP structure finds increasingly more applications on a micro and nanoscale, thermal characteristics become extremely important to understand, because light energy can give rise to thermal fluctuations and, thereby, affect the performance of devices that are integrated to a WGP. For this reason, thermal properties have drawn significant interests in nanoscale optical devices, such as metal and dielectric nanorods^40,41^ and plasmonic nanospheres^42^. Many studies have been performed to understand thermal radiation in a nanoscale gap^43,44^. Near-field heat transfer was investigated between gratings^45^. Thermal properties of magnetic nanoparticles were simulated for hyperthermia treatment of cancer^46^, while thermal resistance was measured and estimated for tungsten silicide WGPs to be high up to 523 K^47^.

In this paper, we intend to compute and analyze thermal distributions produced by a WGP. The thermal distribution can be far from replicating the distribution of light fields because of finite thermal conductivity and radiation in the microscale range. For applications as a polarizing element in an optical set-up and particularly when a WGP is integrated as a part of a device or a package, e.g., grating wire-grids used as a biosensing substrate on which molecular interactions may be measured^48–51^, the thermal distribution on a WGP may affect the biosensor performance and, therefore, exact understanding of thermal characteristics of a WGP is vital. Furthermore, as we explore thermal extinction between the two polarization components, understanding the thermal characteristics may go beyond WGPs and can be a basis for opening novel applications. Polarization-dependent thermal stimulus may be used to develop a new tool for cell-based assays in biomedical applications using thermal “hot” spots to turn on or off molecular processes or an electronic device that can be thermally switched to control and modulate heat transfer electronically with improved functional characteristics.

## Numerical Method and Model

### Calculation of wave-coupled heat transfer equation

For calculation of thermo-optic characteristics produced by WGP structures, wave-coupled time-dependent heat transfer equation was solved, which is given as follows^52–54^:

4 $${C}_{s}\rho \frac{\partial T}{\partial t}+{\bf{n}}\cdot {\boldsymbol{q}}=\frac{1}{2}{\varepsilon }_{0}\omega Im({\varepsilon }_{r}){|E|}^{2}$$

5 $$-{\bf{n}}\cdot {\boldsymbol{q}}=\nabla \cdot [k\nabla T+h({T}_{amb}-T)+{\varepsilon }_{m}\sigma ({T}_{amb}^{4}-{T}^{4})]$$

6 $${\nabla }^{2}E-\mu \varepsilon \frac{{\partial }^{2}E}{\partial {t}^{2}}=0$$

Eq. (4) is the heat transfer equation with the right-hand side referring to the dissipated heat that is generated by electromagnetic absorption and acts as a heat source. **n** and ***q*** are the normal vector to the lateral plane and heat. The three terms in the del operator on the right-hand side of Eq. (5) represent the heat transfer by conduction, convection, and radiation, respectively. Eq. (6) is the wave equation that describes wave propagation in a medium with ε and *μ* as the medium permittivity and permeability. *C*_*s*_ represents the heat capacity, while *ρ* is the medium density and *k* thermal conductivity. *ω* is the angular frequency of incident light. *h* is the convective heat transfer coefficient and *T*_*amb*_ the ambient temperature. *ε*_*m*_ denotes the emissivity of metal (gold in our case) and *σ* the Stefan–Boltzmann constant. *E*(**r**, t) and *T*(**r**, t) denote the spatial distribution of electric field amplitude and temperature. The wave equation in Eq. (6) was solved by frequency analysis using time-independent method and the heat transfer equation of Eq. (4) by time-dependent analysis. Electromagnetic waves were assumed to have a constant field distribution while heat was spreading, because electromagnetic wave propagation is much faster than heat transfer by conduction. This assumption in thermo-optic calculations leads to quasi-static solutions that are in effect much simpler to attain than fully time-dependent approaches.

Note that the difference of the incident power from what is dissipated by WGP may cause imperfect matching between optical calculation and that of heat transfer equation. Furthermore, it is difficult to apply boundary conditions to semi-infinite structures, such as grating, particularly in the case of heat transfer equation, because modeling the heat transferred between cells is tricky when a heat source is in contact with the boundary. These issues were addressed by performing aperiodic calculation on WGP structures, instead of considering wave and heat transfer equation separately over the entire structure which may require heavy memory consumption^55,56^.

### Numerical methods

Far-field characteristics of a WGP were calculated with rigorous-coupled wave analysis (RCWA) using 40 spatial harmonics. For wave optics calculation of optical characteristics, 2D finite element method (FEM) on COMSOL™ was used under scattering and periodic boundary condition with triangular meshes of 0.045 to 22.5 nm in size. Periodic calculation using 2D FEM in the far-field was confirmed with RCWA and 3D FEM models and found to be in agreement.

Thermal properties of WGPs were also calculated using FEM which implemented Eqs (4 and 5). FEM calculation was performed with an incident light intensity at 0.1 mW/μm^2^ under scattering boundary conditions. The intensity was taken as a value that may be obtained under typical experimental conditions. Calculation was also performed with 0.01 mW/μm^2^ as incident intensity for comparison. For the calculation of heat transfer equation, we used a heat flux boundary condition with a heat transfer coefficient set at *h* = 5 W/K·m^2^, which is taken from literature^54^, and an external ambient temperature at *T*_*amb*_ = 293.15 K (room temperature). The substrate was assumed to form periodic boundaries in the lateral direction with a heat transfer coefficient at 0 W/K·m^2^ and grating wires as an electromagnetic heat source. The steady-state temperature was measured at the center of wire-grid surface.

### Numerical model for WGP

**Figure 1:**
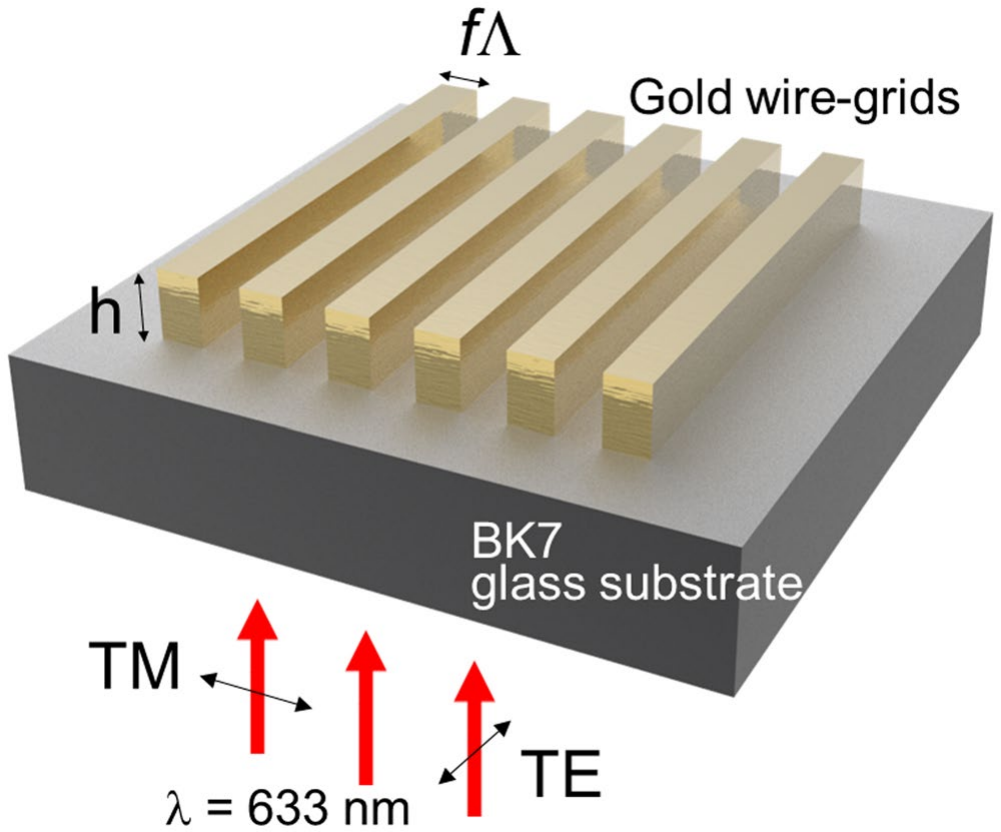
Schematic illustration of a WGP numerical model with wire-grid period *Λ*, fill factor *f*, and height *h*. The direction of electric field oscillation for TM and TE polarized light is also shown.

The schematic of the numerical model employed for computation is presented in Fig. 1. Wire-grid of gold in a rectangular surface profile is assumed on a BK7 glass substrate in air ambiance. Wire-grid periods for numerical computation were varied in a range of *Λ* = 500 nm~1 μm in a step of 100 nm (for RCWA calculation, *Λ* = 400 nm~1 μm with a 10-nm step). The wire-gird thickness or grid height (*h*) and fill factor (*f*) were varied to be *h* = 100~500 nm and *f* = 0.1~0.9. It was assumed that monochromatic plane waves of linear polarization were of normal incidence at λ = 633 nm. Polarization direction corresponding to TM/TE polarized light is also shown in Fig. 1. Throughout computation, refractive index of BK7 glass substrate was set to be *n*_*s*_ = 1.5151. For the simplicity of calculation, the substrate was assumed to be thick by 5*Λ* from 2.5 to 5 μm, an assumption often used in related studies^41,53^. For gold, refractive index was interpolated from reference^57^. We have also assumed that only the zeroth order reflection or transmission is collected by detectors.

### Metrics of WGP performance

As mentioned earlier, the performance of a WGP was assessed with transmittance (*Tr*) and extinction ratio (*ER*). *ER* was represented as *ER* = 10 log(*Tr*^*TM*^/*Tr*^*TE*^) in dB. Similarly, we have evaluated thermal characteristics of a WGP with temperature, *T*^*TM*^ and *T*^*TE*^ (in Kelvin), measured at the center of WGP surface for both polarization components, and also thermal extinction ratio (*ThER*), which we define as the ratio of average local temperature in Celsius for TM to that of TE, i.e.,

7 $$ThER=\frac{{T}^{TM}}{{T}^{TE}}\,\,{\rm{or}}\,\,10\,\mathrm{log}(\frac{{T}^{TM}}{{T}^{TE}})\,{\rm{in}}\,{\rm{dB}}$$

*ThER* reflects on the extinction based on point measurements at the surface. *ThER* should be interpreted simply as a ratio of temperature in Celsius induced with TM polarized light over TE polarization, rather than as a measure of power or heat.

## Results and Discussion

### Far-field optical properties of a WGP

**Figure 2:**
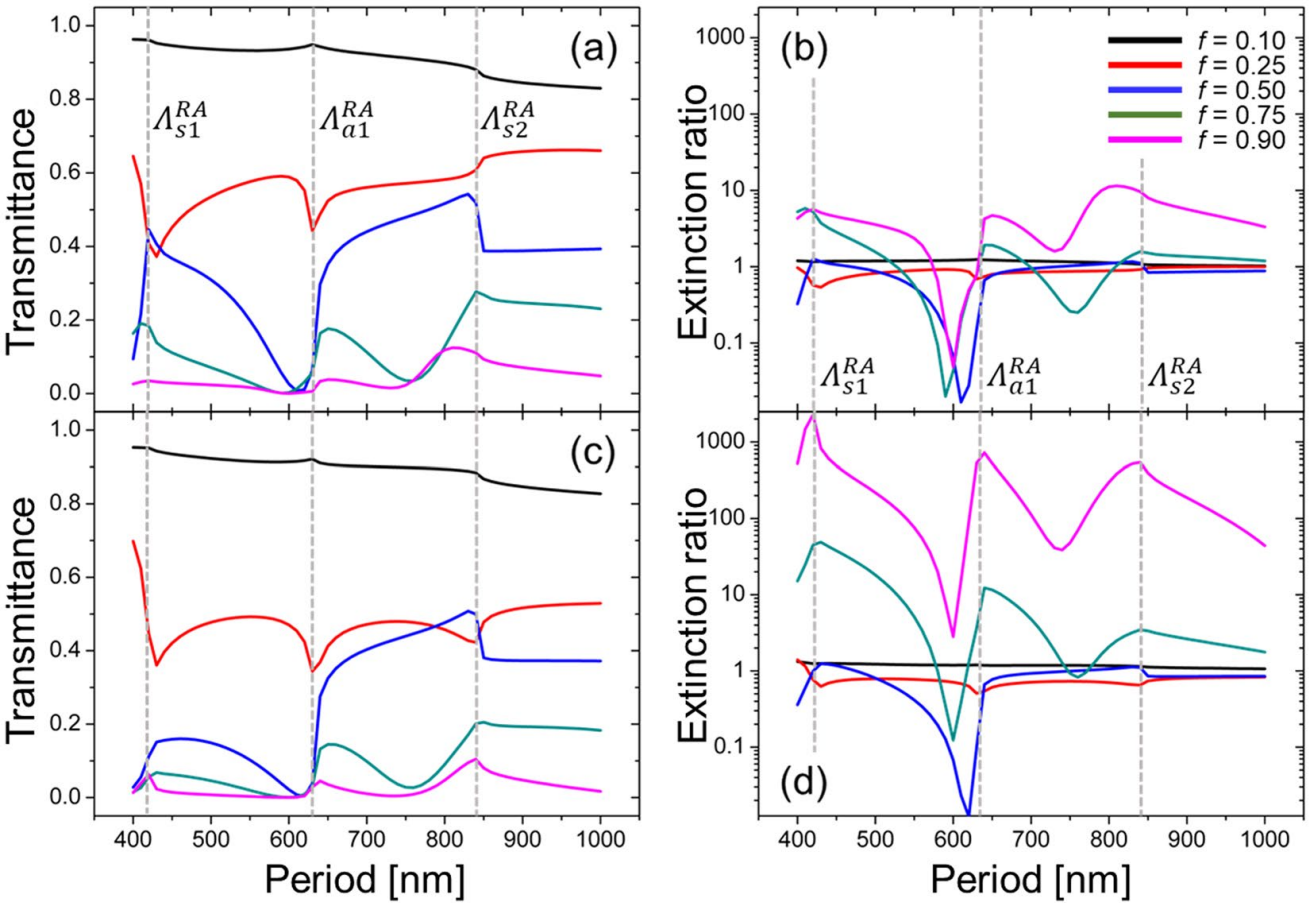
(**a**) Transmittance and (**b**) *ER* of a WGP at normal incidence for Λ = 400~1000 nm with wire-grid height *h* = 100 nm. (**c**) Transmittance and (**d**) *ER* for *h* = 200 nm. The fill factor *f* is varied. ${\Lambda }_{sm}^{RA}$ and ${\Lambda }_{am}^{RA}$ represent the grating period at which Rayleigh anomaly occurs due to *m*-th substrate and ambient modes (marked in gray dotted lines).

Far-field characteristics of a WGP, while well-known, were calculated for confirmation of results, as shown in Fig. 2. Grating equation at normal incidence is given by *k*sinθ_m_ = *mK*_*G*_/*n*_*s*_ for substrate modes and *k*sinθ_m_ = *mK*_*G*_ for ambient modes, where *k* = 2π/*λ* and *K*_*G*_ = 2π/*Λ* represent the incident wave number and the magnitude of grating vector. *m* is an integer used for diffraction orders. θ_m_ denotes the angle of outgoing waves after diffraction. Grating equation shows that Rayleigh anomaly occurs as a result of energy redistribution between diffraction orders for WGP with $\Lambda  > {\Lambda }_{s1}^{RA}$ = *λ*/*n*_*s*_ = 418 nm. In the range of wire-grid periods that we calculate in this work, the energy redistribution takes place at *Λ* = 418 $({\Lambda }_{s1}^{RA})$, 633 $({\Lambda }_{a1}^{RA})$, and 836 nm $({\Lambda }_{s2}^{RA})$. Figure 2 shows transmittance (*Tr*) and extinction (*ER*) produced by a WGP at *h* = 100 and 200 nm. A clear trend presented in Fig. 2 is that transmittance overall decreases, while *ER* increases, with a higher fill factor *f*, i.e., incident light is largely transmitted (reflected) at a low (high) *f* due to the smaller (larger) volume of absorbing metal. For thick WGPs with height on the order of several hundred nanometers, metal-dielectric-metal waveguide modes in the vertical direction may also induce resonances and high absorption with TM polarized light incidence, although the resonance may be weakened at small periods^58^. Maximum *ER* attained with *h* = 200 nm was 2280, although in this case transmittance was low at 6.25%. Similar to the effect of a fill factor, thicker wire-grids tend to increase extinction, while decreasing transmittance.

### Thermal properties of a WGP

**Figure 3:**
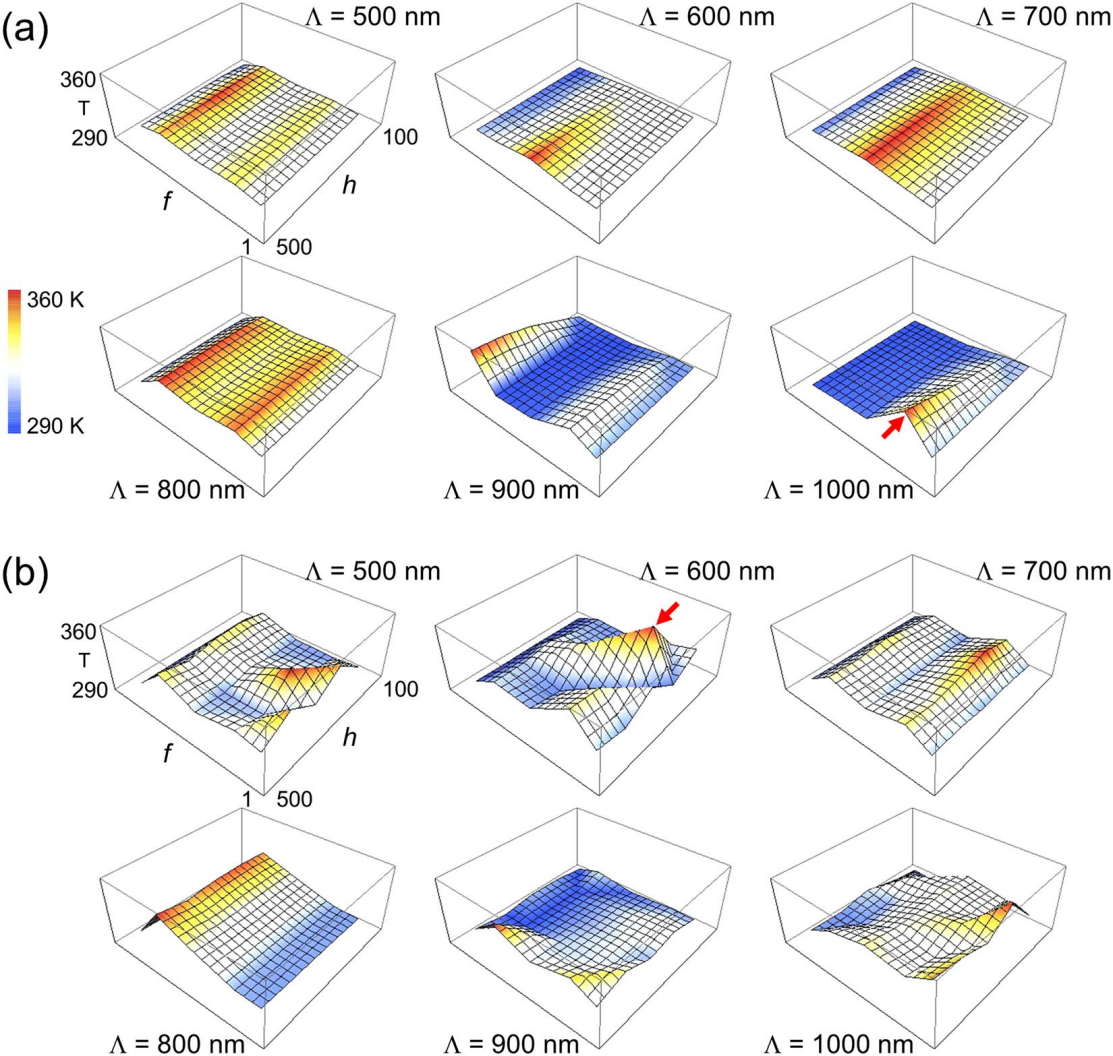
Temperature distribution produced by a WGP for wire period *Λ* = 500 nm~1 μm, fill factor *f* = 0.1~0.9, and grating height *h* = 100~500 nm: (**a**) TE and (**b**) TM polarization. Temperature in absolute temperature (K). Red arrows represent the parameters that produce maximum temperature for TE and TM polarized light: T_max_ = 334.6 K at *Λ* = 1 μm and *h* = 500 nm (TE) vs. 354.5 K at *Λ* = 600 nm and *h* = 200 nm (TM). For both cases, *f* = 0.75.

Figure 3 presents the equilibrium temperature calculated at the center of a WGP for TE and TM polarized light. The temperature induced by TM polarization is overall significantly higher than for TE polarized light with a maximum as high as T = 354.5 K when *Λ* = 600 nm, *f* = 0.75, and *h* = 200 nm. For comparison, maximum temperature reached by TE polarized light is T = 334.6 K when *Λ* = 1 μm, *f* = 0.75, and *h* = 500 nm. The parameters with which the highest temperature is produced are marked by arrows in Fig. 3. The distinct temperature characteristics between polarization components of a WGP is associated with the physical origin of heat transfer induced by TE and TM polarized light. For TE polarization, conductive heat transfer by way of Joule heating that is produced by significant light absorption is dominant. In the case of TM polarization, radiative heat transfer by plasmon-polaron resonant modes dominates, where polaron mode is coupled to plasmon states created by momentum-matching with incident photon in metallic wire-grids. While polaron-plasmon coupling in split-ring resonator (SRR) structure was reported previously^59^, WGPs are presumed to make a more significant temperature difference between TE and TM polarization components than SRRs due to the inherent polarimetric extinction. Heat spreading takes place through ambient air and glass substrate largely by conduction and radiative wave propagation and, to a lesser extent, excitation of surface plasmon polariton modes on the wire-grid/glass substrate interface^60,61^. Thermal fluctuating currents may produce net emission, although its mean is zero. The effect compared to the ambience is estimated to be minor in terms of autocorrelation, according to the fluctuation-dissipation theorem^62^.

Trivariate linear regression analysis was performed with period, height, and fill factor of wire-grids as independent variables. Table 1 shows that a linear model fits better to TM polarization with higher correlation over TE polarization, although it may well be a weak predictor of parameter-dependence of temperature, and suggests the fill factor to be the strongest parameter that affects temperature. For TE polarized light, the effect of wire-grid height is almost as important as that of fill factor. Temperature is positively correlated with a fill factor *f*, presumably because of heat flux associated with absorption cross-section of metallic wire-grids and due to the excitation of guided modes and surface plasmon when the corrugation depth is increased^63^. This encourages more light absorption and more active heat transfer by thermal conduction at the grid-air interface. For TM polarization, although a large part of energy is transmitted, there is still significant absorption which gives rise to heat transfer by conduction and therefore higher ∂T/∂*f*. In contrast, TE polarized light is largely reflected, rather than absorbed, so that the volume effect represented by the fill factor manifests itself weakly. As a result, ∂T/∂*f* is lower with weaker correlation between the temperature and the fill factor. In fact, the time derivative ∂T/∂*f* is 2.60 (=1090/420) times higher for TM polarized light than TE, indicating that an increase of fill factor leads to a higher temperature rise in the case of TM polarization.

**Table 1:** Temperature derivatives with respect to the geometrical parameters for TE and TM polarization as a result of trivariate linear regression analysis (s.d. n = 150, P-R^2^: Pearson correlation coefficient, and R^2^: coefficient of multiple determination).

	TE polarization		TM polarization	
	Temperature derivative	P-R^2^	Temperature derivative	P-R^2^
∂T/∂*Λ* [K/μm]	4.25 ± 3.37	0.101	6.69 ± 5.03	0.104
∂T/∂*h* [K/μm]	8.54 ± 4.07	0.168	9.91 ± 6.07	0.127
∂T/∂*f* [K/%]	0.0420 ± 0.0193	0.174	0.1090 ± 0.0288	0.296
R^2^	0.0685		0.115	

**Figure 4:**
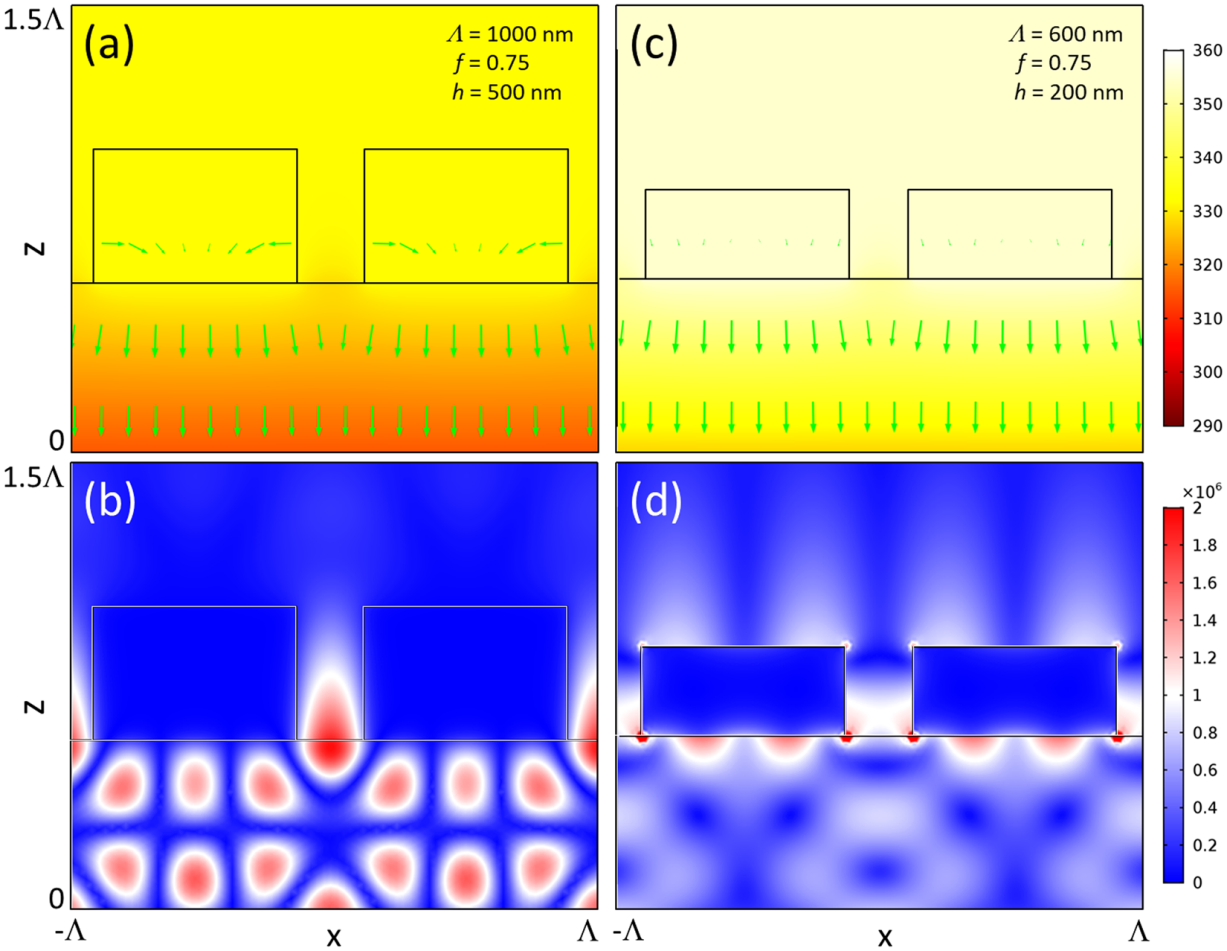
(**a**) Spatial distribution of local temperature and (**b**) electric field amplitude (|*E*^*TE*^|) produced by a WGP: *Λ* = 1000 nm, *f* = 0.75, and *h* = 500 nm with normal light incidence along +z direction in TE polarization (Max: 331.76 K and Min: 331.71 K). (**c**) Temperature and (**d**) electric field (|*E*^*TM*^|): *Λ* = 600 nm, *f* = 0.75, and *h* = 200 nm with TM polarized light (Max: 354.59 K and Min: 354.56 K). Arrows represent the direction and strength of heat flux. Data at 5 μs after light illumination. Temperature in unit of K and electric field in V/m.

The temperature distribution of a WGP with geometric parameters which produce the highest local temperature is shown in Fig. 4. TM-polarized light forms higher temperature distribution along the depth (z) axis and transmissive electric fields through metal wire-grids. Due to more active heat transfer, thermal energy of TM polarization dissipates slightly faster in the depth axis at a rate of *Δ*T/*Δ*z = −73.7 K/mm than that of TE polarization (*Δ*T/*Δ*z = −49.6 K/mm). In general, temperature changes very slowly along the depth axis on the order that is in line with literature^64,65^. It is also interesting that TM polarization induces much less prominent lateral (x) heat flux component than TE polarization.

**Figure 5:**
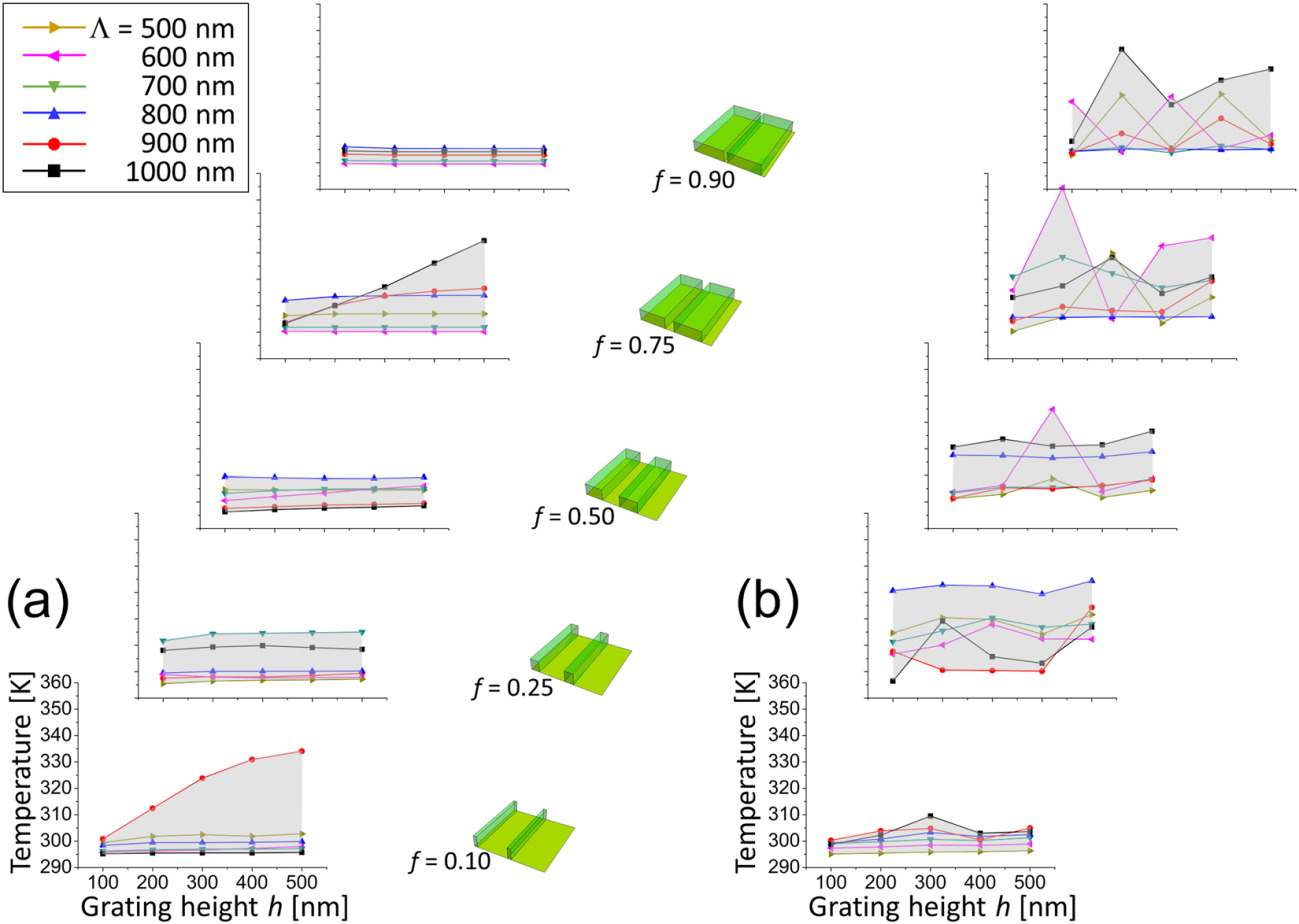
Temperature characteristics produced by a WGP with a fill factor *f* = 0.1~0.9, and grating height *h* = 100~500 nm: (**a**) TE and (**b**) TM polarization. Shaded area represents the temperature variation with respect to wire-grid periods. WGP schematics corresponding to each fill factor are also shown.

Different perspectives can be obtained in Fig. 5, which presents equilibrium temperature in terms of grating height *h* and a fill factor *f*. Clearly, the effect of fill factor appears to prevail in the temperature characteristics while that of grating height is weak. On the other hand, the effect of wire-grid period is relatively inconspicuous in line with what we observed in Fig. 3 and Table 1. Temperature variation is also significantly higher with TM polarized light. For TE polarization, much part of light is absorbed and reflected by wire-grids regardless of the period, therefore the temperature varies with even weaker dependence on wire-grid period. Somewhat erratic behavior of temperature characteristics in the case of TM polarization is presumed to be associated with Rayleigh anomaly. In contrast, a significant portion of TE polarized light is extinguished by metal wires, therefore the effect of Rayleigh anomaly is less severe.

In short, fill factor was found to be the most dominant parameter that affects the temperature. The dependence is stronger in TM polarization than for TE polarized light. Dependence on the wire-grid height and period is weaker over the range that we calculated. TM polarization induces higher variation in the observed temperature with respect to the period.

Aside from the effects of various geometrical parameters, it is worth a note that the near-fields produced by a WGP in air ambience may induce local temperature almost as high as 355 K with an incident light intensity at 0.1 mW/μm^2^. Although not with WGPs, such a high temperature induced in the micro and nanoscale range was observed experimentally, for example, in plasmonic heating^66–68^. Note that heat transfer depends heavily on environmental effects of ambience. Preliminary calculations in water ambience show that the maximum temperature in the steady-state remains lower than in air, because of higher heat capacity of water than that of air (roughly 4.18 vs. 1.00 J). Also, thermal conductivity for water is much higher than air, for example, by more than 20 times under atmospheric pressure and room temperature (0.6096 W/K·m for water vs. 0.02623 W/K·m for air)^69,70^, therefore relaxation of heat takes place on a faster scale.

### Microscale thermal extinction

**Figure 6:**
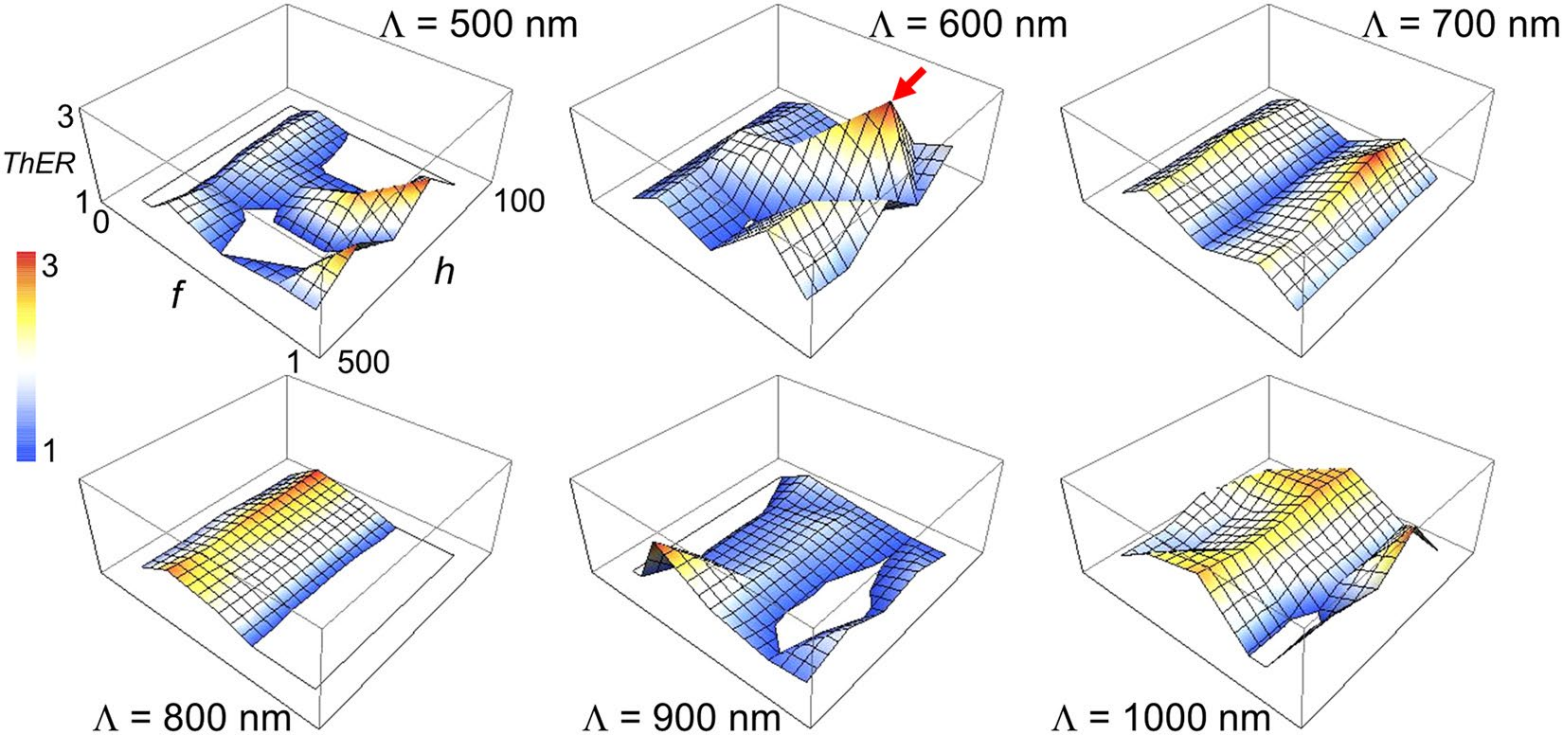
Thermal extinction ratio (*ThER*) of a WGP with a fill factor *f* = 0.1~0.9, and grating height *h* = 100~500 nm for wire-grid period *Λ* = 500 nm~1 μm. Gray represents an area of *ThER* < 1. Highest *ThER* = 3.00 observed at *f* = 0.75 and *h* = 200 nm with *Λ* = 600 nm (marked by arrow).

We now investigate thermal extinction *ThER* achieved by a WGP. Figure 6 shows *ThER* as a function of a fill factor *f* and wire-grid height *h* for wire-grid period *Λ*. The thermal extinction can be important for example, in applications where a polarization-dependent temperature change may induce a process on or off. Gray areas in Fig. 6 represent a region with *ThER* < 1, i.e., the temperature produced by TM polarized light becomes lower than that of TE polarization. Highest extinction was observed as *ThER*_*max*_ = 3.00 (or 4.78 dB) when *Λ* = 600 nm, *f* = 0.75, and *h* = 200 nm. While the maximum thermal extinction ratio may not appear to be significant, the value represents a remarkable temperature difference *ΔT* = 54.3 K, which can be switched on or off by the transition of polarization states. If we consider imaging applications, for example, imaging polarimetry, typical optical input scenes to a WGP consist of mixed polarization components and the results in Fig. 6 suggest that local temperature may vary significantly depending on the input polarization content. On the other hand, this also implies that local temperature may be adjusted with precision by controlling the incident light polarization. Interestingly comparison of Figs 3 and 6 shows that the WGP structure corresponding to the highest optical and thermal extinction is almost identical, suggesting that optical extinction provided by primarily transmissive TM light component gives rise to the thermal extinction.

### Non-equilibrium dynamics

**Figure 7:**
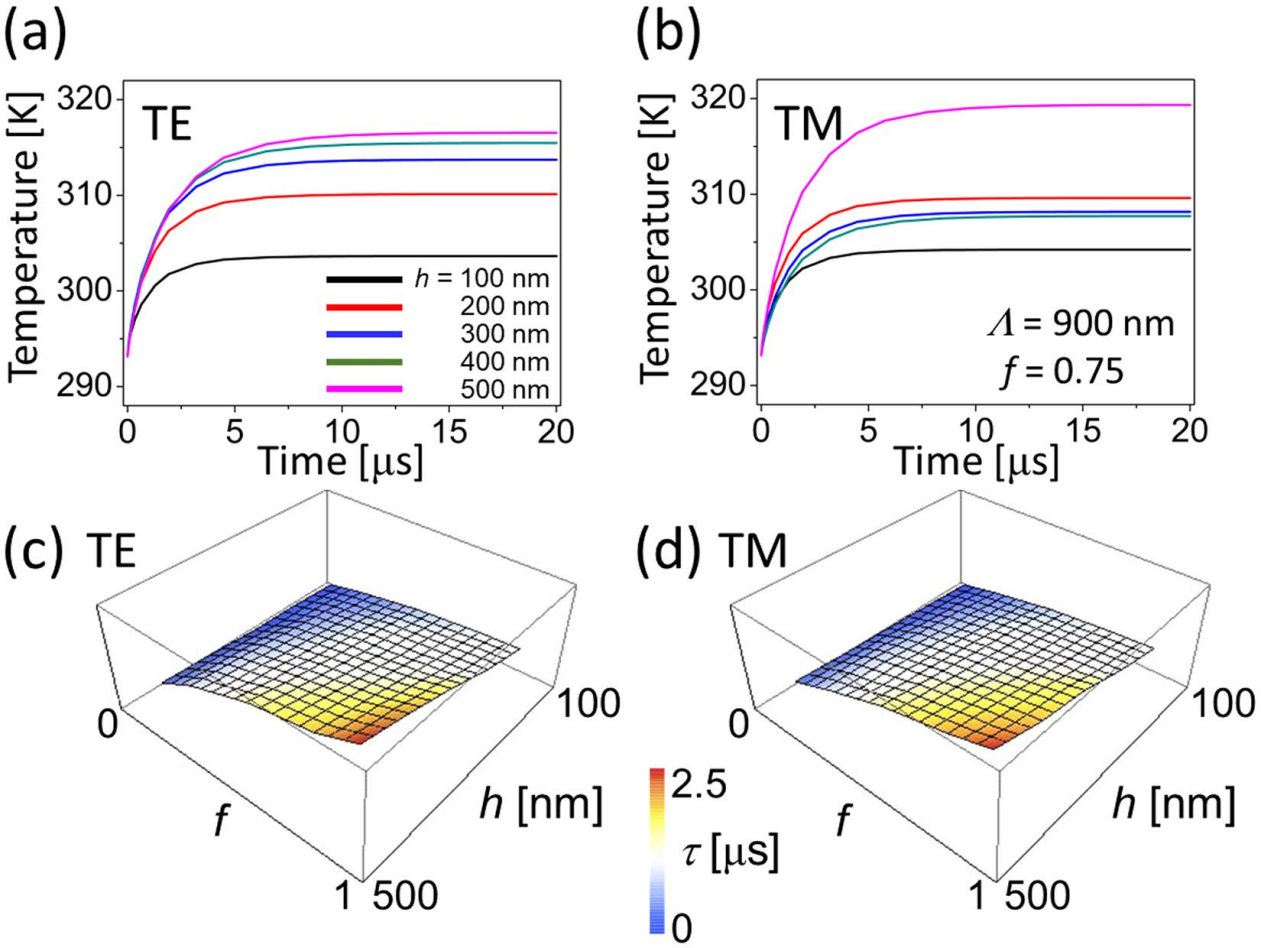
Temperature-time response for a WGP with a period *Λ* = 900 nm (wire-grid height *h* is varied): (**a**) TE and (**b**) TM polarization at *f* = 0.75. Relaxation time (τ) in a 3D plot as a fill factor and a wire-grid height are varied: (**c**) TE and (**d**) TM polarization. Calculation performed up to 20 μs to ensure steady-state.

So far, we have investigated steady-state thermal characteristics of a WGP assuming that heat is dissipated fast enough to reach a steady-state. We now explore thermal relaxation time (τ) that takes for a WGP to reach an equilibrium in the stead-state. The relaxation time was extracted from an exponential fit of temperature-time responses and calculation was performed up to 20 μs to ensure that the steady-state is reached. Within the relaxation time, temperature characteristics may vary significantly from the steady-state. Although the way that the temperature rises presented in the temperature-time response of Fig. 7(a,b) may look different between TE and TM polarization, the relaxation time is surprisingly similar as shown in Fig. 7(c,d). The ratio of relaxation times (τ_TM_/τ_TE_) ranged from 0.857~1.450, i.e., the ratio of relaxation times was found to be largely uniform within 15% and much lower than optical or thermal extinction, because the thermal process by which the steady-state is reached through absorption and conduction is in principle identical for both TE and TM polarization. Overall, the relaxation time was on the order of μs (10^−6^ sec), which agrees well with experimental results observed in comparable structures^52^. The relaxation time varies depending on geometrical parameters. First, thicker wire-grids would make thermal energy transfer difficult and therefore relaxation time longer. On the other hand, while a longer wire-grid period increases the relaxation time, the dependence on the fill factor was relatively weak.

### Effect of light intensity

While all of the above temperature characteristics of a WGP were obtained with 0.1 mW/μm^2^, we have also explored 0.01 mW/μm^2^ as light intensity to investigate the effect of incident power. The highest temperature was calculated to be 299.3 K for TM and 297.3 K for TE polarization, as listed in Table 2. It is suggested that the induced temperature distribution may be in the linear regime in the sense that the temperature rise due to light incidence, which is represented by ΔT (=T_max_ − T_min_), is linearly related to the input intensity. With much higher intensity beyond the order of 10 mW/μm^2^, the temperature increase was found to become sublinear likely due to the effects of nonlinear optics, although more exhaustive investigation is needed to understand the specific nature of the power dependence.

**Table 2:** Temperature characteristics with an incident light intensity at 0.01 and 0.1 mW/μm^2^ (T_max_ and T_min_: highest and lowest temperature, ΔT = T_max_  − T_min_).

Input power [mW/μm^2^]		T_max_/T_min_/ΔT [K]	WGP structure		
			*Λ* [nm]	*f*	*h* [nm]
0.01	TE	297.3/293.4/3.9	1000	0.75	500
	TM	299.3/293.3/6.0	600		200
0.1	TE	334.6/295.2/39.4	1000		500
	TM	354.5/295.1/59.4	600		200

## Concluding Remarks

As a summary, we have investigated microscale thermo-optic properties of a WGP. It was found that local temperature may rise up to almost 355 K over 80 °C under the given parameters with an incident power at 0.1 mW/μm^2^. Although the dependence was not strong enough to generalize for an arbitrary WGP structure, TM polarization overall induces higher temperature than TE polarized light, with increased temperature at higher fill factor. In the parameter space that we have considered, a WGP offers significant thermal extinction by 3 times (or 4.78 dB) in terms of *ThER* for *Λ* = 600 nm, *f* = 0.75, and *h* = 200 nm, which represents more than 54 °C in temperature difference by polarization switching between TE and TM. We have also explored relaxation dynamics in a WGP. The results may be useful for understanding thermal characteristics of a WGP and providing thermal extinction for applications that may require polarization-dependent switching of heat transfer.
